# Effect of Simvastatin on Arterial Stiffness in Patients with Statin Myalgia

**DOI:** 10.1155/2015/351059

**Published:** 2015-07-15

**Authors:** Kevin D. Ballard, Lindsay Lorson, C. Michael White, Paul D. Thompson, Beth A. Taylor

**Affiliations:** ^1^Division of Cardiology, Henry Low Heart Center, Hartford Hospital, Hartford, CT 06102, USA; ^2^Department of Kinesiology and Health, Miami University, Oxford, OH 45056, USA; ^3^Department of Health Sciences and Nursing, University of Hartford, West Hartford, CT 06117, USA

## Abstract

Statins reduce arterial stiffness but are also associated with mild muscle complaints. It is unclear whether individuals with muscle symptoms experience the same vascular benefit or whether statins affect striated and smooth muscle cells differently. We examined the effect of simvastatin treatment on arterial stiffness in patients who did versus those who did not exhibit muscle symptoms. Patients with a history of statin-related muscle complaints (*n* = 115) completed an 8 wk randomized, double-blind, cross-over trial of daily simvastatin 20 mg and placebo. Serum lipids and pulse wave velocity (PWV) were assessed before and after each treatment. Muscle symptoms with daily simvastatin treatment were reported by 38 patients (33%). Compared to baseline, central PWV decreased (*P* = 0.01) following simvastatin treatment but not placebo (drug ∗ time interaction: *P* = 0.047). Changes in central PWV with simvastatin treatment were not influenced by myalgia status or time on simvastatin (*P* ≥ 0.15). Change in central PWV after simvastatin treatment was inversely correlated with age (*r* = −0.207, *P* = 0.030), suggesting that advancing age is associated with enhanced statin-mediated arterial destiffening. In patients with a history of statin-related muscle complaints, the development of myalgia with short-term simvastatin treatment did not attenuate the improvement in arterial stiffness.

## 1. Introduction

Hydroxy-methylglutaryl-coenzyme A (HMG-CoA) reductase inhibitors (statins) inhibit mevalonate production, effectively reducing low-density lipoprotein- (LDL-) cholesterol concentrations. Additionally, statins are associated with multiple vascular benefits [1, 2] that may contribute to reduced cardiovascular disease (CVD) morbidity and mortality [3–5]. Reductions in central arterial stiffness (assessed noninvasively by arterial pulse wave velocity (PWV)) with statin use [6–10] represent one such vascular benefit.

Statins are well-tolerated but can produce mild muscle complaints such as muscle pain (myalgia), cramps, weakness, and stiffness. It is not known whether patients who exhibit muscle symptoms with statin use demonstrate the same improvement in central arterial stiffness as nonmyalgic patients. Observation of unchanged central arterial stiffness with statin use in myalgic patients might support a generalized effect on muscle cells (both striated and smooth) by which statins influence skeletal muscle stiffness and fatigue. The present investigation examined the effect of simvastatin treatment on PWV in patients who did versus those who did not exhibit statin-associated muscle symptoms during the run-in phase of the Co-Enzyme Q10 in Statin Myopathy study, of which the methods have been described in detail [11, 12].

## 2. Methods

### 2.1. Study Design

Men and women ≥20 yrs of age with a history of muscle complaints during statin treatment were recruited and enrolled into a randomized, double-blind, crossover, run-in trial of simvastatin 20 mg/d or placebo to confirm statin myalgia [11, 12]. Following discontinuation of cholesterol medications for 4 wk, subjects were treated for 8 wks or until myalgia persisted for 1 wk or became intolerable. Subjects then underwent 4 wk washout period and received the alternative treatment for 8 wks or until myalgia persisted for 1 wk or became intolerable. Subjects were queried about muscle complaints using the Short-Form Brief Pain Inventory [13] at each study visit and were contacted weekly by study personnel to inquire about muscle complaints. Plasma samples were collected and arterial PWV measured at the beginning and end of each treatment phase. In order to maintain study blinding, plasma samples were analyzed for lipids upon study completion (Clinical Laboratory Partners, Hartford Hospital). The Institutional Review Board at Hartford Hospital approved the study and the study was monitored by a Data Safety Monitoring Board. This study was registered at ClinicalTrials.gov (NCT01140308).

### 2.2. Confirmation of Myalgia

Subjects were defined as myalgic if they developed muscle symptoms during simvastatin treatment only. If a participant developed muscle symptoms during both simvastatin and placebo treatments or reported no muscle symptoms during simvastatin treatment, they were considered nonmyalgic [11, 12].

### 2.3. Arterial Stiffness Assessment

Following a 10 min supine rest period, measurements of pulse wave analysis and pulse wave velocity (PWV) were performed with the SphygmoCor CPV Central Blood Pressure/Pulse Wave Velocity System (AtCor Medical, Sydney, Australia). Multiple pulse waveforms of the right carotid and right femoral artery were recorded sequentially by applanation tonometry to determine central PWV. The transit time was determined by measuring the distance between the points of measurement of the carotid and femoral pulses, recorded by taking measurements on the surface of the body from the suprasternal notch to the point where the right carotid pulse was found and from the suprasternal notch to the right femoral pulse via the umbilicus. Peripheral PWV was measured as the transit time between the right radial and the right femoral artery waveforms. Pulse waveforms obtained over a 10 sec period at the right radial artery were used to compute a corresponding central waveform using a validated mathematical transformation. Central systolic (CSBP) and diastolic blood pressure (CDBP), augmentation pressure (AP), and augmentation index (AIx) are reported as pulse wave analysis parameters.

### 2.4. Statistical Analyses

Data (means ± SD) were analyzed by SPSS Version 19.0 (SPSS Inc., Chicago, IL, USA). Prior to all analyses, normality of data was assessed using Shapiro-Wilk's *W*-test. Initial analyses were performed using 3-way repeated measures ANOVA to examine effects due to drug, time, and gender. All drug*∗*time*∗*gender interactions were not significant (all *P* > 0.05) and thus male and female data are combined. Independent samples *t*-test, Mann-Whitney *U* test, or chi-square test was performed to examine differences in participant characteristics between myalgics and nonmyalgics at baseline. Three-way repeated measures ANCOVA was used to examine main and interactive effects due to drug, time, and myalgia status, covarying for time on drug. A Student's paired *t*-test or Wilcoxon signed rank test was used to evaluate comparisons between study drugs at baseline and between the baseline and the end of the trial within each treatment group in the case of a statistically significant drug*∗*time interaction. Linear regression analyses were performed to evaluate if baseline variables or the magnitude of change in plasma lipids predicted changes in arterial stiffness. Further models were run controlling for sex and age. An *α*-level of *P* ≤ 0.05 was considered statistically significant for all analyses.

## 3. Results

### 3.1. Participant Characteristics

Characteristics of participants who did (*n* = 38, or 33%) versus those who did not (*n* = 77) meet the study definition for myalgia [11, 12] are summarized in Table 1. Compared to nonmyalgics, participants with myalgia were heavier and were treated with simvastatin for a shorter duration (*P* < 0.05). Five nonmyalgic participants were on simvastatin treatment for >8 wks (range = 8.3–11.7 wks) due to reported missed doses or antibiotic treatment.

### 3.2. Changes in Plasma Lipids

Simvastatin treatment produced the expected reductions in plasma total cholesterol, LDL cholesterol, and triglycerides (Table 2). Neither myalgia status (*P* ≥ 0.31) nor time on simvastatin (*P* ≥ 0.17) influenced lipid changes.

### 3.3. Changes in Arterial Stiffness

**Figure 1:**
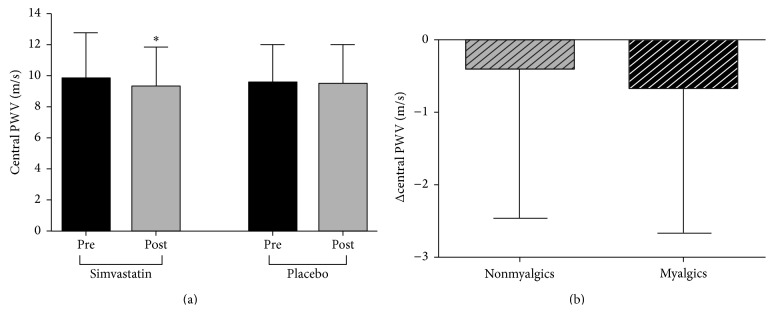
Data are means ± SD. (a) Central pulse wave velocity (PWV) before (Pre) and after (Post) 8 wks of simvastatin (*n* = 100) or placebo treatment (*n* = 100); ^*∗*^ *P* < 0.05 compared to Pre within a group. (b) Changes (Post-Pre) in central PWV of participants who completed the simvastatin intervention with no development of statin-related muscle symptoms (nonmyalgics; *n* = 76) compared with those who reported muscle symptoms with daily simvastatin treatment (myalgics; *n* = 34); *P* = 0.51 between groups.

No differences in arterial stiffness measures were observed between study drug groups at baseline (Table 3). Only central PWV showed a significant drug*∗*time interaction (*P* = 0.047). Compared to baseline (9.8 ± 2.9 m/s), central PWV decreased following simvastatin treatment (9.3 ± 2.4 m/s; *P* = 0.01) but not placebo (Figure 1(a)). Neither myalgia status (*P* = 0.62) (Figure 1(b)) nor time on simvastatin (*P* = 0.15) influenced central PWV suggesting that the development of myalgia did not attenuate the improvement in central arterial stiffness with simvastatin treatment.

**Figure 2:**
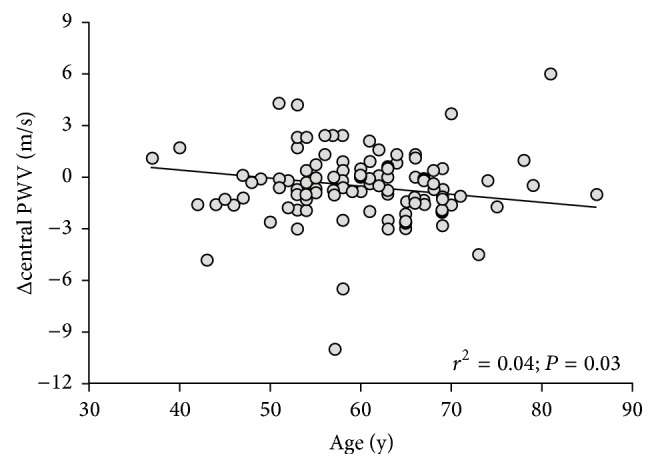
Relationship between changes (Post-Pre) in central pulse wave velocity (PWV) and age in simvastatin-treated participants (*n* = 110). Δ = absolute change from before to after the treatment intervention.

Age was inversely correlated with the change in central PWV after simvastatin treatment (*r* = −0.207, *R* ^2^ = 0.043, *P* = 0.03) (Figure 2), suggesting that advancing age is associated with enhanced statin-mediated arterial destiffening. The magnitude of change in central PWV after simvastatin treatment was positively correlated with changes in plasma LDL cholesterol concentrations when sex and age were controlled (*r* = 0.336, *R* ^2^ = 0.113, *P* < 0.01).

## 4. Discussion

The exact mechanism(s) by which statins induce muscle complaints is not known [14, 15]. Depletion of intramuscular metabolites produced by the mevalonate pathway with subsequent increase in cytosolic calcium and activation of mitochondrial-mediated skeletal myocyte apoptosis may contribute to statin myopathy [16]. Findings from the present study of improved central arterial stiffness with simvastatin treatment, irrespective of the development of myalgia, suggest that the mechanism(s) underlying statin muscle symptoms is specific to skeletal myocytes and does not impact vascular smooth muscle with statin therapy. Thus, these data do not support a generalized mechanism of statin myalgia (i.e., impacting both skeletal and smooth muscle cells) as one would expect a differential impact of simvastatin therapy on arterial stiffness in myalgics versus nonmyalgics to occur.

Increased central PWV independently predicts CVD and all-cause mortality [17], suggesting that interventions that reduce central arterial stiffness are clinically important. Central arterial destiffening with statin therapy has been observed in some [6–10] but not all [18–21] studies, an inconsistency potentially explained by differential effects of statin type on arterial stiffness [22] and/or heterogeneity between PWV protocols [23]. Findings from the present study are the first to show similar reductions in arterial stiffness in patients who did versus those who did not exhibit muscle symptoms with statin use as prior studies [6–10] did not assess muscle complaints.

The precise mechanism(s) underlying statin-mediated reductions in central arterial stiffness [6–10] remains unclear. Improvements in endothelial function [1] or suppression of sympathetic neural activity [24, 25] have been proposed as mechanisms by which statins reduce vascular smooth muscle tone and central arterial stiffness in humans. While defining the mechanism was beyond the scope of the present study, our observation of decreased central PWV with simvastatin treatment within a relatively short period of time (≤8 wks) suggests that statins mediate arterial destiffening through functional rather than structural mechanisms. Furthermore, our finding of a direct relationship between the degree of LDL cholesterol lowering and the magnitude of reduction in central PWV with simvastatin treatment suggests a contribution of lipid lowering to central arterial destiffening. In contrast, previous studies in healthy adults have reported reductions in central PWV to occur independently of lipid lowering [7]. Increased statin use [26] warrants future clinical studies aimed at determining the mechanism(s) responsible for statin-mediated central arterial destiffening and whether improvements in PWV with chronic statin therapy translate into lower CVD risk.

Our observation of an inverse relationship between age and reductions in central PWV after simvastatin treatment suggests that the beneficial effects of statins on arterial stiffness may be enhanced with aging. Statins may more beneficially impact older arteries through their antioxidant actions [1] based upon evidence that oxidative stress plays a pathophysiological role in the age-associated reductions in large artery compliance in humans [27]. Limited evidence exists regarding age-dependent pleiotropic effects of statins, an area that warrants future investigation due to the increasing proportion of older adults [28, 29].

Limitations of the present study, including statin type, dose, and treatment duration, have been described [12]. Nevertheless, our observation of reduced central arterial stiffness with simvastatin treatment for ≤8 wks, independent of the development of muscle symptoms, is strengthened by our large sample size and cross-over study design.

## 5. Conclusions

In summary, findings from this randomized, double-blind, cross-over study show that simvastatin reduces central arterial stiffness in patients with a history of statin-associated muscle complaints. Central PWV was reduced similarly in patients who did versus those who did not exhibit muscle symptoms despite differences in time on simvastatin. Reductions in central arterial stiffness occurred independently of the development of statin-related muscle symptoms, suggesting that statins do not differentially impact arterial stiffness in patients reporting skeletal muscle symptoms with statin treatment. Thus, our data do not support a generalized effect on muscle cells as a mechanism by which statins influence skeletal muscle stiffness and fatigue.

## Figures and Tables

**Table 1:** Baseline characteristics of participants by myalgia status^a,b,c^.

	Nonmyalgic sample (*n* = 77)	Myalgic sample (*n* = 38)
Age, y	60.9 ± 8.5	59.1 ± 10.0
Men, *n* (%)	42 (55%)	25 (66%)
Height, m	1.7 ± 0.1	1.7 ± 0.1
Weight, kg	80.8 ± 15.8	87.4 ± 17.9^*∗*^
BMI, kg/m^2^	27.7 ± 4.2	29.8 ± 5.2^*∗*^
SBP, mmHg	123.4 ± 13.7	121.5 ± 13.1
DBP, mmHg	74.9 ± 6.4	75.1 ± 6.9
Time on simvastatin, wks	7.3 ± 1.6	3.8 ± 2.0^*∗*^
TC, mmol/L	6.56 ± 1.14	6.35 ± 1.04
LDL-C, mmol/L	4.23 ± 1.00	4.11 ± 0.80
HDL-C, mmol/L	1.41 ± 0.38	1.33 ± 0.39
TG, mmol/L	4.51 ± 2.85	4.57 ± 2.98

^a^Data are means ± SD or proportions. ^b^BMI, body mass index; DBP, diastolic blood pressure; LDL-C, low-density lipoprotein cholesterol; HDL-C, high-density lipoprotein cholesterol; SBP, systolic blood pressure; TC, total cholesterol; TG, triglycerides. ^c^Plasma lipid levels assessed prior to initiating simvastatin or placebo treatment did not differ (*P* ≥ 0.23) and therefore were averaged. ^*∗*^ *P* ≤ 0.05 between samples.

**Table 2:** Plasma lipid changes by drug assignment and myalgia status^a,b^.

	Nonmyalgic sample (*n* = 77)		Myalgic sample (*n* = 38)	
	Simvastatin	Placebo	Simvastatin	Placebo
ΔTC, mmol/L	−1.50 ± 0.84^*∗*^	−0.04 ± 0.58	−1.67 ± 0.61^*∗*^	−0.03 ± 0.70
ΔLDL-C, mmol/L	−1.44 ± 0.67^*∗*^	−0.01 ± 0.50	−1.51 ± 0.60^*∗*^	−0.14 ± 0.60
ΔHDL-C, mmol/L	0.08 ± 0.18^*∗*^	−0.01 ± 0.15	0.04 ± 0.18	0.02 ± 0.14
ΔTG, mmol/L	−0.65 ± 2.47^*∗*^	0.04 ± 1.66	−1.00 ± 2.50^*∗*^	0.48 ± 2.73

^a^Data are means ± SD; Δ = absolute change from before to after the treatment intervention.

^b^LDL-C, low-density lipoprotein cholesterol; HDL-C, high-density lipoprotein cholesterol; TC, total cholesterol; TG, triglycerides. ^*∗*^ *P* < 0.01 from baseline. There were no differences (*P* ≥ 0.18) between nonmyalgic and myalgic participants within a treatment group.

**Table 3:** Arterial stiffness changes by drug assignment^a,b^.

	Simvastatin		Placebo	
	Baseline	Study end	Baseline	Study end
Peripheral PWV, m/s	10.3 ± 1.9	10.4 ± 1.8	10.7 ± 2.5	10.2 ± 1.7
Central SBP, mmHg	115.1 ± 14.6	112.7 ± 11.6	113.3 ± 12.2	112.1 ± 13.3
Central DBP, mmHg	75.7 ± 6.9	75.1 ± 7.6	74.9 ± 6.9	74.5 ± 8.9
AP, mmHg	11.9 ± 6.4	11.4 ± 5.0	12.1 ± 5.2	10.9 ± 5.2
AIx, %	23.4 ± 9.4	23.5 ± 8.9	24.9 ± 9.4	23.0 ± 8.9

^a^Data are means ± SD (*n* = 96). ^b^AIx, augmentation index; AP, augmentation pressure; DBP, diastolic blood pressure; SBP, systolic blood pressure; PWV, pulse wave velocity. There were no differences at baseline (*P* ≥ 0.07) between nonmyalgic and myalgic participants within a treatment group.
